# Early tissue and healing responses after maxillary sinus augmentation using horizontal platelet rich fibrin bone blocks

**DOI:** 10.1186/s12903-023-03228-z

**Published:** 2023-08-24

**Authors:** Shimin Yu, Yinping Tian BD, Yan Wei BD, Mengge Feng BD, Sensen Li BMed, Guoyong Tong BMed, Zhouqing Yu BMed, Richard J. Miron, Yufeng Zhang, Zaibo Yang, Yulan Wang

**Affiliations:** 1 State Key Laboratory of Oral & Maxillofacial Reconstruction and Regeneration, Key Laboratory of Oral Biomedicine Ministry of Education, Hubei Key Laboratory of Stomatology, School & Hospital of Stomatology, Wuhan University, Wuhan, 430079 China; 2 Department of Stomatology, The Central Hospital of Enshi Tujia and Miao Autonomous Perfecture, Enshi, 445000 China; 3 Department of Periodontology, School of Dental Medicine, University of Bern, Bern, Switzerland; 4 Medical Research Institute, School of Medicine, Wuhan University, Wuhan, 430071 China

**Keywords:** Horizontal platelet-rich fibrin, Inflammatory cells, Maxillary sinus augmentation, Vascularization

## Abstract

**Background:**

The effects of horizontal platelet-rich fibrin (H-PRF) bone block on the healing and immune response during sinus augmentation have not been fully investigated histologically at early time points.

**Methods:**

Eighteenth male New Zealand white rabbits underwent bilateral sinus augmentation and were divided into two groups: deproteinized bovine bone mineral (DBBM) alone and H-PRF + DBBM (H-PRF bone block) group. Maxilla samples were collected at 3, 7 and 14 days post sinus augmentation procedures and analyzed using histological staining for the number of inflammatory cells, new blood vessels and evidence for early osteoclast bone turnover/remodeling. Furthermore, the effects of H-PRF bone blocks on the migration of osteoblasts and THP-1 macrophages were evaluated using a Transwell assay in vitro.

**Results:**

A higher number of immune cells were found in the H-PRF bone block group at 3 and 7 days post-surgery when compared to the DBBM alone group,most notably in the regions close to the mucosal lining and bone plates. Furthermore, a significantly greater number of new blood vessel formations and early signs of osteoclast development were found in the H-PRF bone block group at 14 days. The in vitro transwell assay further confirmed that culture medium from H-PRF bone block markedly promote the migration of osteoblasts and THP-1 macrophages.

**Conclusions:**

The findings from this study have shown that H-PRF bone block is capable of increasing early immune cell infiltration leading to the acceleration of neovascularization and speeding the process of bone metabolism in vivo following maxillary sinus grafting with DBBM.

## Background

Bone resorption brought on by sinus pneumatization following tooth loss or inflammatory conditions requires bone grafting prior to dental implant therapy [1–3]. Maxillary sinus augmentation procedures using a lateral window approach have been well described in the literature and have shown to be an effective modality in rebuilding sufficient bone volume for future implant placement [4–6]. A variety of bone substitute materials, including autologous bone, xenogeneic bone, and demineralized or mineralized allogeneic bone, have been utilized [1, 5]. One proposed advantage of the deproteinized bovine bone mineral (DBBM) which has been widely used for such applications is that it possesses a better ability to maintain long-term bone stability, even years after grafting has taken place [7–9].

Recently it has been suggested that under certain clinical parameters, bone formation can be achieved during maxillary sinus augmentation procedures without bone grafting during simultaneous implant placement [10]. Despite the fact that maxillary sinus grafting without the use of bone grafts showed slightly lower implant survival rates when compared to grafting with a bone graft [10], these results highlight the importance of blood clot formation during the space maintenance phase and confirm their ability to induce bone regeneration within the maxillary sinus, even without the presence of a bone graft.

Horizontal Platelet-rich fibrin (H-PRF) is a novel biomaterial derived from harvesting blood from the patient prior to surgery with a simplified preparation process that does not require any chemical additives. PRF, unlike the first version platelet rich plasma which required anticoagulants, results in slow and natural polymerization of fibrin similar to a blood clot but with higher bioactivity and structural integrity as it contains a higher concentration of living cells, autologous growth factors, and a three-dimensional fibrin network [11]. Thus, many clinicians have proposed combining particulate bone grafts with PRF to favor better handling of the graft and by providing the early blood-derived proteins and cells required for healing [12–14]. A recent study found that by combining solid H-PRF clots with bone particles and liquid H-PRF, a moldable ‘sticky bone’ block was prepared with quick solidification, excellent mechanical strength properties and greater biological/regenerative properties [15]. The H-PRF bone block has also been favored since it improves the handling characteristics during surgery compared to particulate bone substitutes alone, especially for maxillary sinus grafting [15, 16].

One of the proposed mechanisms by which H-PRF has been shown to improve healing is derived from its ability to attract and provide binding sites to blood cells, including immune cells and osteoblasts originating from peripheral blood [17].

It is known that an initial immune response induced by tissue damage right after surgery is necessary for healing, composed of the innate immune system, including neutrophils, monocytes, and macrophages, having an anabolic function by then recruiting osteogenic cell [18]. Although the early inflammatory state is very crucial for wound healing, there are no in vivo studies investigating the early tissue response of PRF during the early healing phases of maxillary sinus grafting, with most studies concentrating on long-term bone volume at 4 to 8-week time points [10, 19–23]. We hypothesized that H-PRF, as a biological scaffold enriched with leukocytes and platelets, could accelerate the process of inflammatory enrichment and thus accelerate wound healing. For these reasons, the present study focused on the early tissue and healing responses during maxillary sinus grafting using H-PRF bone blocks compared to DBBM alone using a rabbit model, focusing on the immune cell infiltration patterns and new vessel formation.

## Materials and methods

### Animal model design

The rabbit model and experimental designs followed the ARRIVE guidelines for animal research. All the procedures carried out with animals were approved by the Ethics Committee at the School of Stomatology, Wuhan University, People’s Republic of China (B52/2020). Considering that the rabbit model permits the use of both maxillary sinuses, the study was designed as a randomized split-mouth study. The number of animals required was estimated on the basis of previous study which comparing the mean extent of bone formation at the 7 days after tricalcium phosphate, PRF + tricalcium phosphate were applied to sinus elevation in rabbits [24]. PASS 15.0 software (NCSS, USA) was used to calculate the sample size, and the Two-Sample T-Test Assuming Equal Variance module was adopted, with the standard deviation set at 5%, the statistical significance was set as α = 0.05, the statistical efficacy as 0.9, and the component ratio as 1:1. The minimum sample size was needed for study was six in each group at each period of time: 3, 7 and 14 days. All rabbits underwent bilateral maxillary sinus augmentation, and the left and right maxillary sinuses were randomly assigned as follows: control group = DBBM (Geistlich Bio-Oss®, Geistlich Pharma North America, Inc., Wolhusen, Switzerland) and the test group = H-PRF + DBBM (bone block) group. The animals were randomly allocated to their healing time point, and 36 samples were collected, resulting in six data for each group at each time point (n = 6). Thus, a total of eighteen male adult New Zealand rabbits, mean weight of 2.5 to 3.0 Kg, aged 6 months were obtained and kept at a constant temperature to acclimate to the facility for about one week prior to the surgery. Animals were housed in separate cages under standard laboratory conditions, with free access to water and a standard diet.

### The preparation of H-PRF

For the collection of venous blood of rabbits, the hair on the cranial head and ears were shaved and the skin was sterilized locally. After injecting 20% urethane (Urethane, Sinopharm Chemical Reagent Co., Ltd., Ningbo, China) at approximately 5 ml kg -1 body weight intravenously into the ear’s lateral marginal vein, two tubes of 6 ml whole blood were drawn from the other ear side. The glass tubes and plastic tubes (Plasmatrident, Weiyin Technology Co., Ltd., Wuhan, China) were used to prepare solid H-PRF and liquid H-PRF respectively and immediately centrifuged at 700 g for 8 min using a horizontal centrifuge (Eppendorf 5702, Hamburg, Germany) based on previous studies [25–27].

As for migration assays, peripheral blood samples were collected from three healthy volunteers aged 18–30 years with no anticoagulant or antibiotic usage for at least 3 months before blood collection after informed consents. All procedures performed in this study involving human participants were performed in accordance with the 1964 Declaration of Helsinki and the ethical standards of the Ethics Committee at the School of Stomatology, Wuhan University, People’s Republic of China (B52/2020). Written informed consents. Four 10-ml glass tubes (Plasmatrident, Weiyin Technology Co., Ltd., Wuhan, China) were used to produce solid H-PRF and two 10-ml plastic tubes (Plasmatrident, Weiyin Technology Co., Ltd., Wuhan, China) were used to produce liquid H-PRF.

After the blood collection, two different materials of tubes were centrifuged at 700 g for 8 min using a horizontal centrifuge (Eppendorf 5702, Hamburg, Germany) based on previous studies [25–27]. For solid H-PRF, the clots were cut into small fragments after carefully removing the red blood cell layer. The liquid H-PRF above the red blood cell layer was collected using a syringe.

### The preparation of H-PRF bone block

After collection of H-PRF fragments and liquid H-PRF, 0.1 g of DBBM was mixed with the solid H-PRF fragments and 0.5 ml of liquid PRF was added to the mixture. During the 2 min solidification period, the H-PRF bone block was prepared into cylindrical shapes.

### Surgical protocols

The sinus lift procedure was performed by two operator who have been trained to perform the operation according to previous studies [15, 28]. Briefly, a straight incision of about 1 cm was performed along the sagittal midline on the nasal bone, and a full-thickness flap was elevated. A circular window was prepared using a 6-mm diameter trephine (MICA kitTM, Megagen Implant Co., Ltd., Seoul, Korea) on each side of the maxillary sinus under saline-solution irrigation. The resected bony plate was then carefully removed, and the Schneiderian membrane was elevated to form a maxillary sinus bone graft site. Each sinus was randomly assigned to either the test or the control groups. For the control group, 0.1 g of DBBM was grafted in the sinus opening. The H-PRF bone block, made by mixing the same amount of DBBM (0.1 g) with the solid H-PRF fragments and 0.5 ml liquid H-PRF, was grafted in the prepared sinus opening after solidification as the treatment group.

After completing the grafting process, the bony plate was put back into place to cover the window, then the periosteum and skin were sutured closed with 4 − 0 polyglactin 910 suture (Monocryl, AgnTho’s, Lidingo, Sweden). All rabbits were medicated with 30, 000 U /kg of penicillin potassium (Penicillin potassium, Huachu, Zhengzhou, China) for 3 days by intramuscular administration. Group assignment was performed using random number generation. The animals were allocated to the healing time point randomly. The rabbits were sacrificed at 3, 7 and 14 days by intramuscularly injected with an overdose of anesthetics of Urethane (Sinopharm Chemical Reagent Co., Ltd., Ningbo, China) and pentobarbital sodium (Shanghai Pharma New Asia Pharmaceutical, China). The augmented sinuses segmented from the cranium were fixed with 10% neutral buffered formalin.

### Histological procedures and histomorphometric analyses

All specimens were decalcified using 10% ethylene diamine tetra acetic acid solution for 1 month and then rinsed with distilled water for 24 h. After decalcification, all specimens were dehydrated with gradient ethanol and subsequently embedded in paraffin as described previously [29]. Paraffin samples were sectioned coronally along the center of the sinuses serially with a thickness of 4 μm thick using a semi-automatic vibrating blade microtome and mounted on polyline-coated microscope slides. Three rectangular regions of interest (ROIs; size: 1 mm × 1 mm) were defined: (i) close to the antrostomy (Bony plate), (ii) in the center of the grafts (Inside), (iii) subjacent to the sinus mucosa (Mucosa). About 15 serial sections were harvested from each sinus for the experiments. Two sections from the 15 serial sections were stained for each kind of staining and 1 was chosen for quantitative analysis. Then 6 representative fields in each ROI under ×20 magnification were chosen for quantitative analysis in each section. The number of immune cells and vessels in the sinus areas was investigated using hematoxylin and eosin (H&E) staining. The existence and number of osteoclasts at fourteen days were investigated using TRAP (TRAP staining kit, Solarbio, Beijing, China) staining according to the manufacturer’s protocol. The number of immune cells, vessels and osteoclasts was counted and was conducted on ten consecutive sections of each specimen. Six representative fields (1024 × 1536 pixels) were identified and averaged among each section under ×20 magnification. A blinded, experienced examiner conducted histomorphometric measurements.

### Cell culture

Human osteoblasts (hFOBs) and THP-1 (human acute monocytic leukemia cell line) were purchased from Shanghai Yu Bo Biotech Co., Ltd. and cultured in a humidified atmosphere at 37 °C in DMEM for hFOBs and RPMI-1640 medium for THP-1 (Gibco-Invitrogen, Carlsbad, CA, USA) supplemented with 10% fetal bovine serum (FBS, Beyotime Biotech) and 1% antibiotics (100 U/ml penicillin G, 100 𝜇g/ml streptomycin, HyClone, Thermo Fisher Scientific Inc) respectively. All cells were cultured at 37 °C in a humidified 5% CO2 and 95% air environment. To generate THP-1-derived macrophages, 1 × 10^6^ THP-1 cells were treated with 100 ng/ml PMA for 48 h and adherent cells were collected for further research.

### Migration assay

Transwell chambers with a pore size of 8 𝜇m (Corning Costar, USA) were used to quantify the migration ability of osteoblasts when cultured with conditioned medium from each of the groups of bone blocks. In order to avoid the effect of DBBM itself on cells, culture media from the H-PRF bone block group and the DBBM group was utilized.

0.1 g of DBBM and H-PRF bone block were inserted into the bottom of the 24-well plate. For the preparation of the extracts, 0.1 g DBBM and H-PRF bone block made with 0.1 g DBBM were transferred to a 6-well plate and incubated with 5 ml DMEM and 5 ml RPMI-1640 supplemented or with 10% FBS and 1% antibiotics (Gibco, Thermo Fisher Scientific) in a 37 °C incubator for 3 days. Then, the medium was filtered and stored at 4 °C as PRF conditioned media for future experiments.

Using a standard Transwell assay protocol, the hFOBs and THP-1 were starved for 12 h (control medium supplemented with 1% FBS) in the upper compartment (10^5^ cells/100 𝜇l/well), then 500 𝜇L of the conditioned medium containing 10% FBS was added to the lower wells. After 24 h of migration, the cells were fixed with 4% formaldehyde for 15 min and then stained with 0.1% crystal violet solution (Good Bio Technology Co., Ltd, Wuhan, China) for 15 min. Then, phosphate buffered saline (PBS, HyClone/Thermo Fisher Scientific) was used to rinse the samples three times. The migrated cells in the lower chamber were then captured under the Olympus DP71 microscope (Olympus Co., JP) and counted with ImageJ software (ImageJ v2.1, National Institutes of Health, Bethesda, MD). All experiments were repeated three times independently.

### Statistical assessment

Each of the histological experiments was performed with at least six replicates in all experiments. The statistical analysis was performed using the GraphPad Prism software 8.0.2. Statistical analysis was made by 1- or 2-way ANOVA and Student’s t test. All data were displayed as the mean ± standard deviation. Data are graphed as mean ± SD. ∗p < 0.05, ∗∗p < 0.01, and ∗∗∗p < 0.001 are considered statistically significant.

## Results

### General findings

Slight soft tissue edema was observed during the sinus augmentation surgery and the early healing period. No other adverse events or complications such as Schneiderian membrane perforation or wound dehiscence were noted in either group.

### Histomorphometric analysis

#### H-PRF bone block promoted the aggregation of immune cells

**Fig. 1:**
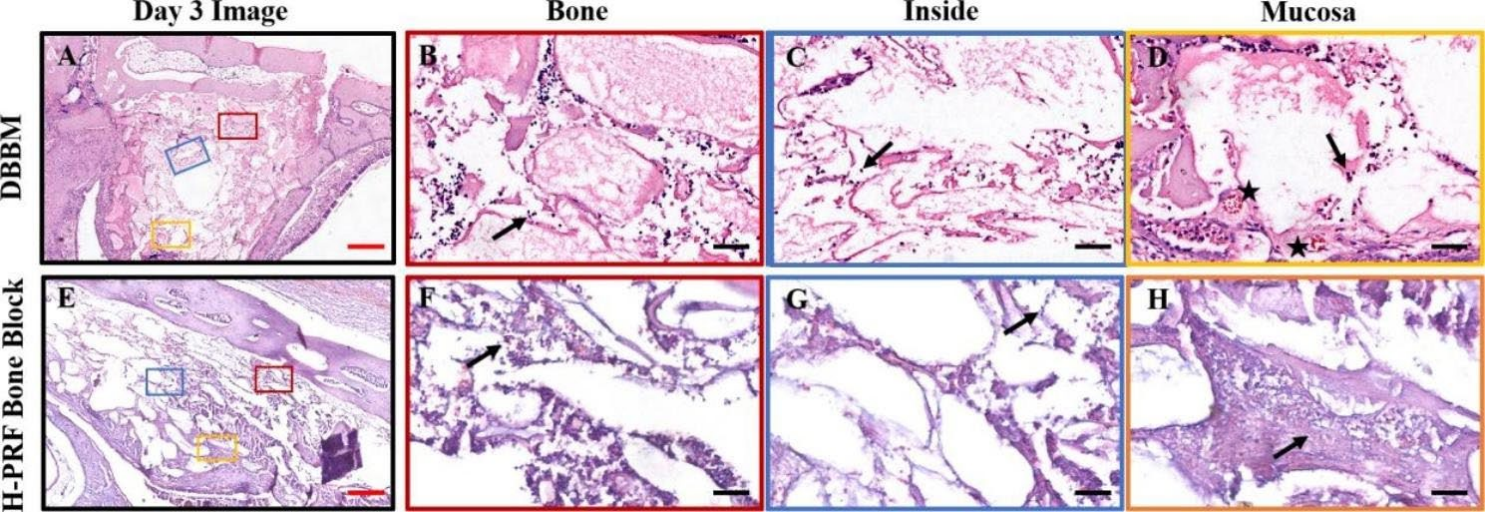
Photomicrographs of hematoxylin and eosin (H&E) staining sections of bioptic samples derived three days post-surgery. Three different areas were chosen for comparison: Bone (area close to the antrostomy); Inside (in the center of the material); Mucosa (area nera the Schneiderian membrane); Black arrows: the inflammatory cells appear as dark blue dots; Asterisks: blood vessels. Scale bar in A,E = 2 mm; B,C,D,F,G,H = 50 μm

**Fig. 2:**
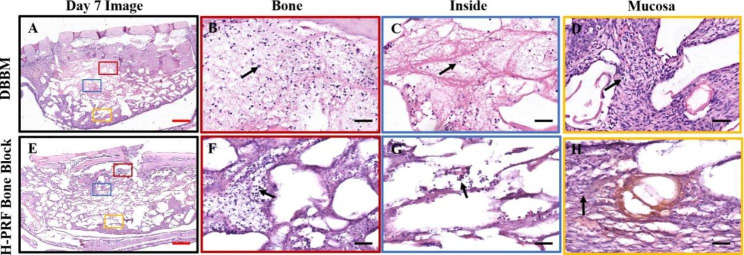
Photomicrographs of hematoxylin and eosin (H&E) staining sections of bioptic samples derived seven days post-surgery. Three different areas were chosen for comparison: Bone (area close to the antrostomy); Inside (in the center of the material); Mucosa (area nera the Schneiderian membrane); Black arrows: the inflammatory cells appear as dark blue dots; Asterisks: blood vessels. Scale bar in A,E = 2 mm; B,C,D,F,G,H = 50 μm

**Fig. 3:**
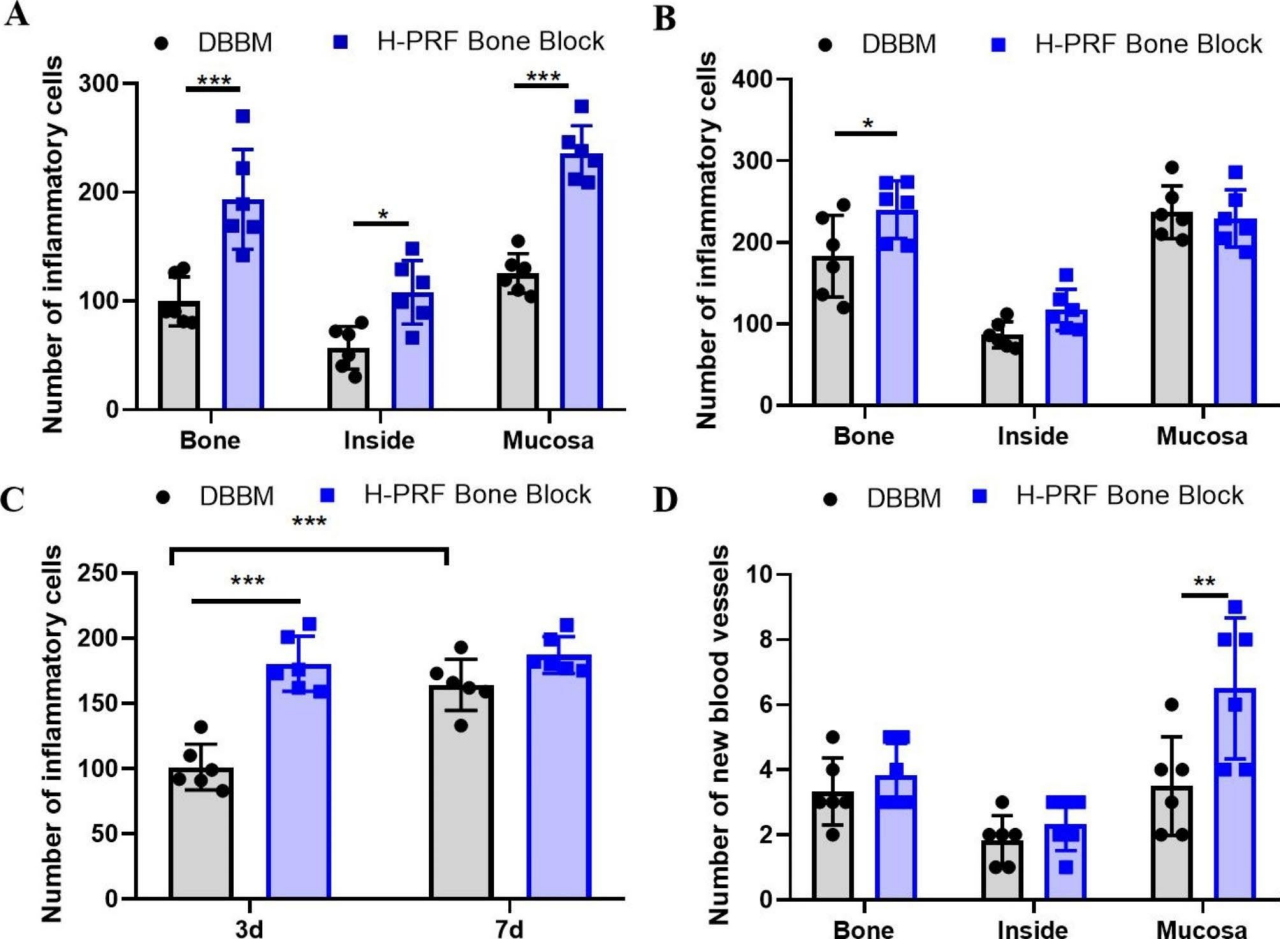
Quantitative analysis of inflammatory cells. **A**. Three days post-surgery (n = 6); **B**. Seven days post-surgery (n = 6); (C) Comparison of total inflammatory cells on three days and seven days post-surgery (n = 6); (D) Neovascularization score 14 days post-surgery (n = 6). ns: not statistically significant versus control group, *p < 0.05, **p < 0.01, and ***p < 0.001

The HE staining was performed to investigate the immune cell accumulation in different areas (Figs. 1 and 2). The center of the samples presented with a general loose texture owing to the early time points with inflammatory cells stained as dark blue cells at 3 and 7 days. The H-PRF bone block group displayed a statistically significantly greater number of immune cells when compared to the control group, especially when the region of interest was harvested from the bone plate and mucosa (*p* < 0.001)(Fig. 3A.B). The total number of immune cells surrounding the H-PRF bone block displayed the greatest difference with the control group at day 3. The number of immune cells in the DBBM alone group gradually increased from days 3–7 while it was observed that the H-PRF bone block group did not see such an increase and instead early angiogenesis was beginning to be observed (Fig. 3C).

**Fig. 4:**
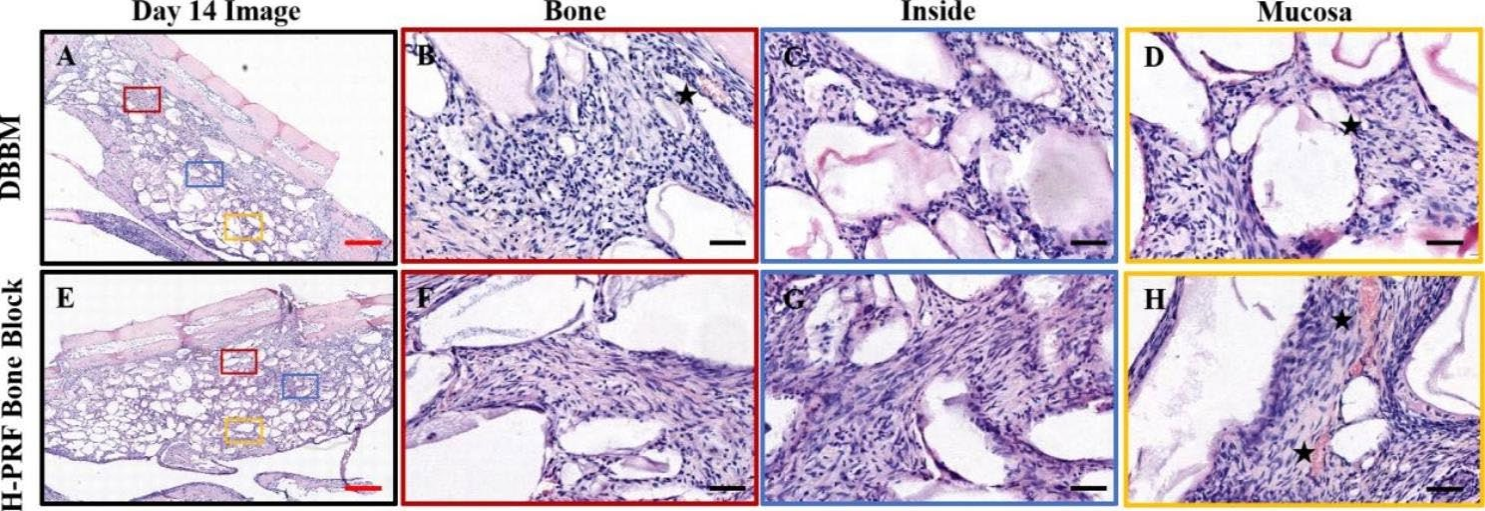
Photomicrographs of hematoxylin and eosin (H&E) staining sections of bioptic samples derived fourteenth days post-surgery. Three different areas were chosen for comparison: Bone (area close to the antrostomy); Inside (in the center of the material); Mucosa (area nera the Schneiderian membrane); Asterisks: blood vessels. Scale bar in A,E = 2 mm; B,C,D,F,G,H = 50 μm

### H-PRF bone blocks promoted early angiogenesis

The neo-angiogenesis in the sinus was investigated by H&E staining (Fig. 4). In different areas, the number of new vessels was higher in the H-PRF bone block group than in the control group, and the difference was statistically significant near the mucosa. The average number of new vessels near the mucosa was 6.500 ± 2.168 in the H-PRF bone block group and 3.500 ± 1.517 in the DBBM group at 14 days respectively, suggesting that the addition of H-PRF significantly improved new angiogenesis(Fig. 3D).

### In vitro migration assay

**Fig. 5:**
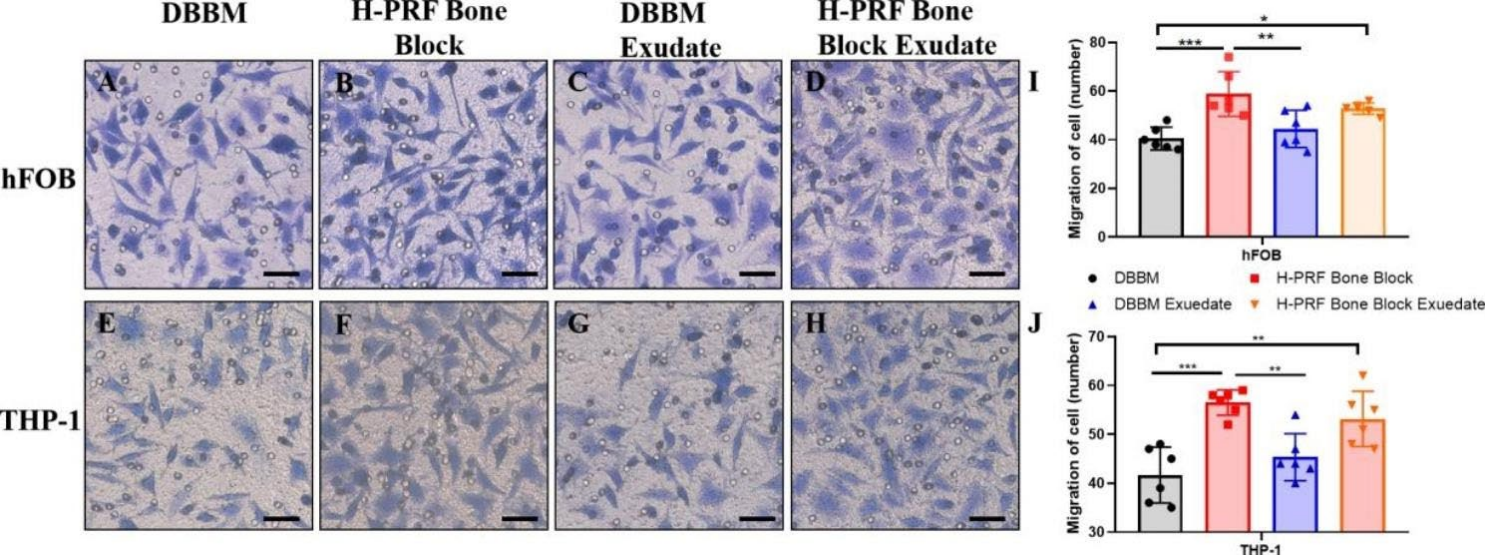
Migration assay of osteoblasts and THP-1 cells using a transwell assay. A-D. Light microscopic images of osteoblast migration; E-H. Light microscopic images of THP-1 migration; I. Quantitative analysis of relative number of osteoblasts; J. Quantitative analysis of the relative number of THP-1 cells. Samples were performed in triplicate with 6 independent experiments. ns: not statistically significant versus control group, *p < 0.05, **p < 0.01, and ***p < 0.001

Since the above histological results showed significant infiltration of immune cells in the H-PRF bone block group, we hypothesized that there might be two reasons for this. One part of the inflammatory cells might be innate immune cells entrapped in the H-PRF scaffold, and the other may be related to the ability of H-PRF to recruit cells from neighboring tissues. Therefore, an in vitro migration assay was used to observe whether the growth factors found in H-PRF bone blocks could recruit immune cells and/or osteoblasts. The results demonstrated that the groups incubated with media from the H-PRF bone block showed more remarkable migration of both cell types when compared to DBBM alone (Fig. 5).

### Evaluation of early bone remodeling

**Fig. 6:**
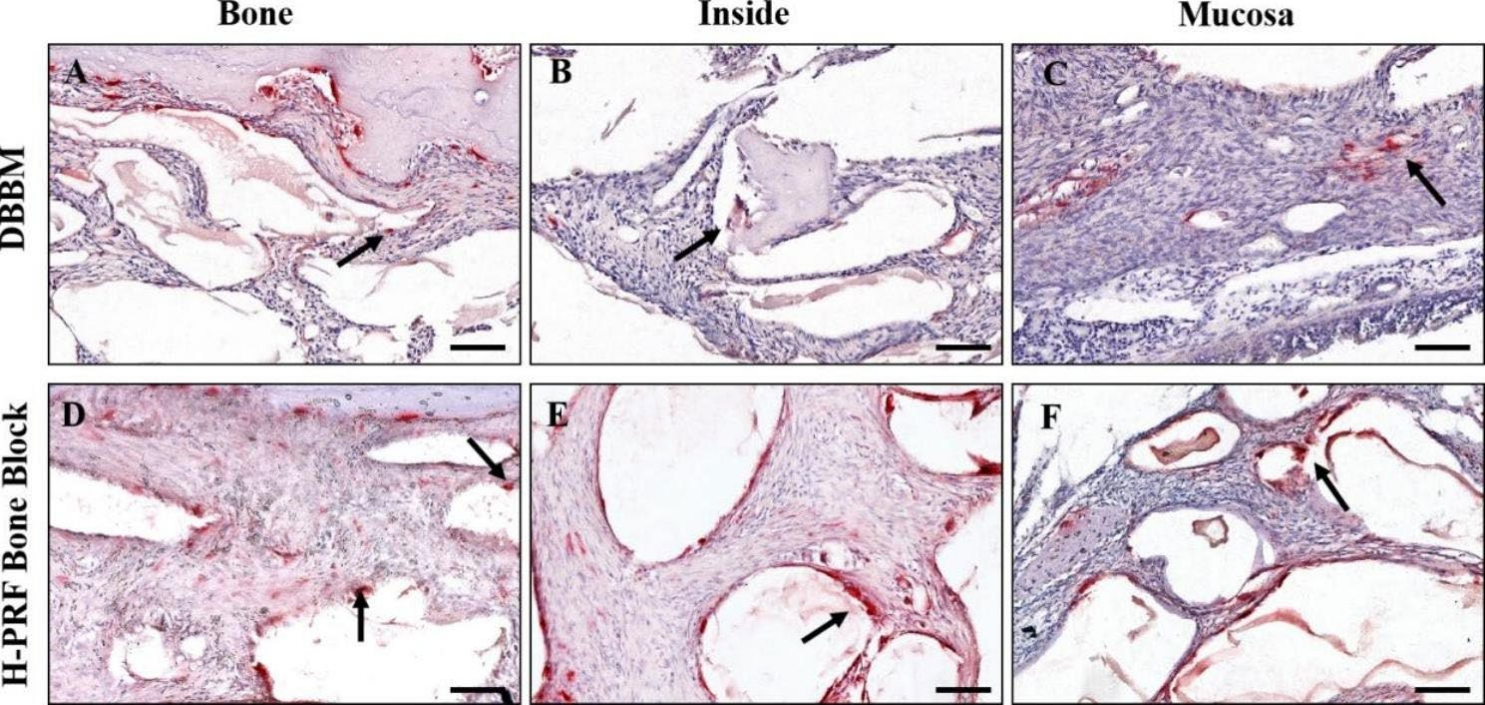
Photomicrographs using tartrate-resistant acid phosphatase (TRAP)-stained osteoclasts (arrows) at fourteen days. **A**,**D**. near the bone plate; **B**,**E.** the inside of augmentation region; **C**,**F**. near mucosa region (×20)

**Fig. 7:**
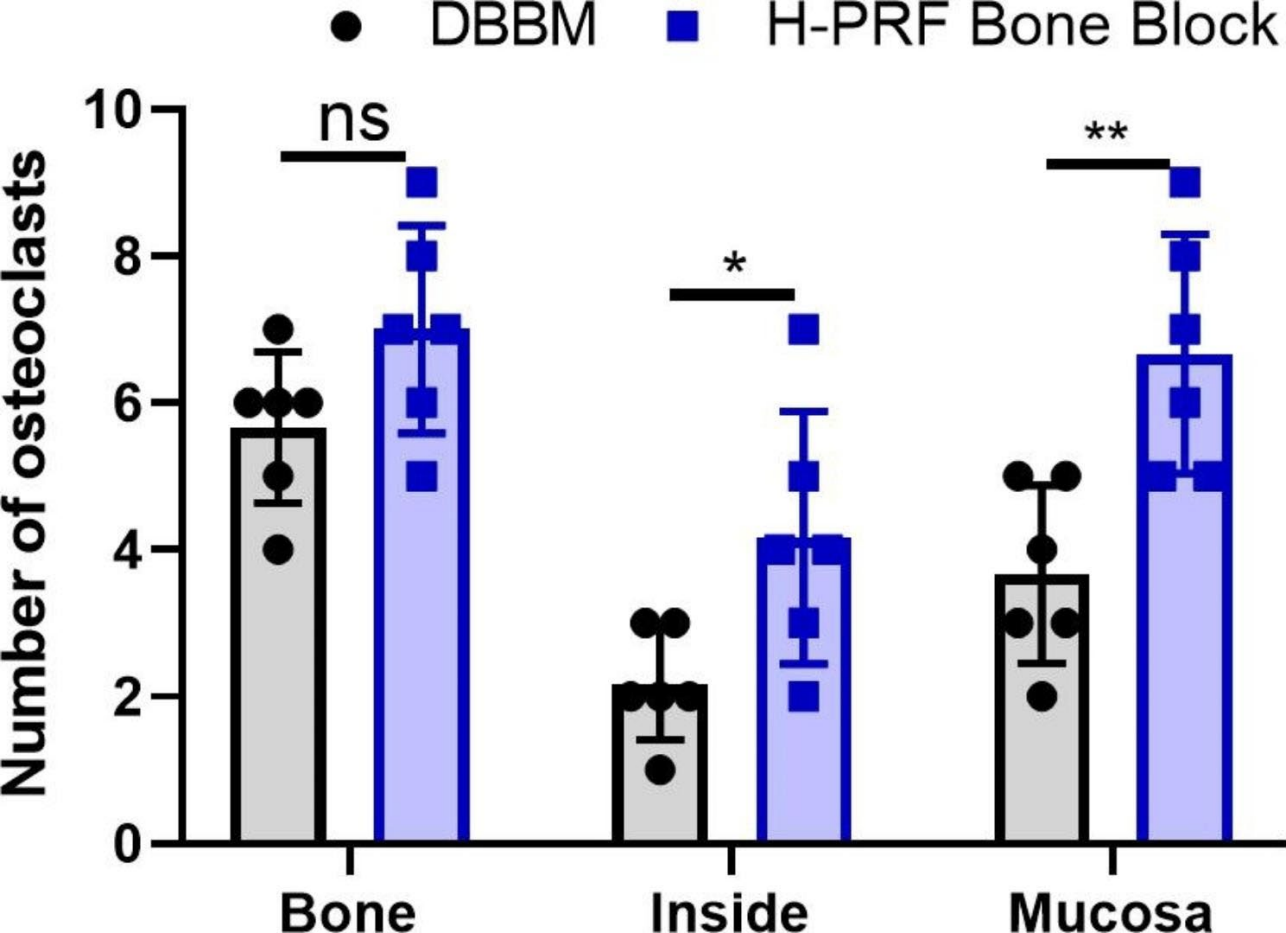
Histomorphometric measurements of the number of osteoclasts at fourteen days in different areas. (n = 6). ns: not statistically significant versus control group, *p < 0.05 and ***p < 0.001

The tartrate-resistant acid phosphatase (TRAP)-stained osteoclasts (arrows) are presented in Fig. 6. The number of osteoclasts at the region near the bone plates as well as inside and near the mucosa was 5.667 ± 1.003, 2.167 ± 0.753, 3.667 ± 1.211 in the DBBM group and higher (7.001 ± 1.414, 4.167 ± 1.722 and 6.667 ± 1.633) in the test group respectively (Fig. 7). Thus, the H-PRF bone block group showed nearly a 2-fold increase in early osteoclast activity, suggesting faster bone remodeling at earlier time points when H-PRF was included in bone grafting of the sinus.

## Discussion

Maxillary sinus augmentation has always been a hotspot in the treatment of patients with posterior bone deficiency. The mechanisms that contribute to the effectiveness and speed of bone regeneration are heterogeneous. Every healing process begins phylogenetically with an inflammatory reaction which must be tightly controlled [30]. Healing of bone defects consists of four steps: hematoma formation, fibrocartilaginous callus formation, bony callus formation and bone remodeling [31]. The early tissue response after grafting is crucial including the rapid infiltration of immune cells [30]. Bone regeneration during maxillary sinus lift is a complex process. As many studies have focused on the ultimate osteogenic effect, few have focused on the early healing stage [32, 33]. Therefore, a rabbit model was used to evaluate the potential effects of H-PRF bone blocks on the early stages of healing following sinus augmentation. It has been shown that the rabbit sinus model is ideal since it is relatively easy to enter the cavity and the sinus structure even the air changes in the nasal cavity are similar to those in humans [31, 34]. Another advantage of the rabbit model is that the auricular vein can easily be utilized to gain sufficient blood for the production of H-PRF. Noteworthy, the concentration of platelets in normal rabbit blood is higher than in humans, which may potentially speed healing in this rabbit model [35].

Hematoxylin and eosin (H&E) staining was then utilized to investigate the number of immune cells and the number of new blood vessels. The histological results showed significant infiltration of immune cells in the H-PRF bone block group at day 3, while the number of immune cells in the DBBM alone group gradually increased and reached a similar degree by day 7. The earlier onset of inflammation also led to increased vascularization. Our study showed that the H-PRF bone block group had significantly higher new vessel formation when compared to DBBM alone, which is hypothesized to result from the increased release of VEGF from H-PRF.

We hypothesized that many of the immune cells might be recruited from neighboring tissues as opposed to solely found in the H-PRF during its preparation [36, 37]. Results from in vitro migration experiments showed that conditioned media from the H-PRF bone block promoted the migration of THP-1 and hFOBs, which might benefit bone regeneration and remodeling subsequently. The reason may be due to the platelets, lymphocytes and related growth factors such as vascular endothelial growth factor (VEGF), transforming growth factor beta 1(TGF-β), and platelet derived growth factors (PDGF) entrapped in H-PRF have all been shown to promote the migration and proliferation of various cell types [38, 39].

In addition to observing early vascularization and inflammatory infiltration, we supplemented osteogenic assessment at 14 days. The activation and complex interaction between angiogenic and osteogenic pathways are crucial during bone repair and remodeling [40]. Several studies have demonstrated that angiogenesis precedes the onset of osteogenesis [41, 42]. The blood vessels of bone tissue can transport minerals and other supplement sources, represent the physical structures around which bone deposition start [43]. Blood vessels also release paracrine signals such as VEGF to modulate the growth, differentiation and regeneration of different cell types [42].

Bone remodeling is a complex process that involves both osteoblasts and osteoclasts. TRAP-staining was used to visualize early osteoclast formation as few studies on PRF have investigated early osteoclast formation. Osteoclasts were found near the sinus membrane and bone walls, consistent with the resulting early increase in inflammatory cells. According to this study, H-PRF promoted the early recruitment of various cell types including immune cells, the formation of new blood vessels, and the formation of osteoclasts during the early stages of sinus augmentation.

These results indicate that PRF can promote the regeneration process of maxillary sinus augmentation. Extended to the clinic, it can remind clinicians that through simple clinical blood collection and centrifugal operation, the plastic and biologically active ‘bone block’ with active cells and growth factors in the patient’s own blood can be constructed with little trauma. On the one hand, compared with ordinary bone graft materials, it has a certain plasticity and mechanical strength, is easier to fill, but also easy to maintain height. On the other hand, this study showed that it can accelerate inflammation, promote vascularization and osteoclast activity. It is beneficial to the subsequent osteogenesis process. For some patients, faster and better bone regeneration can lead to better implant areas, and the importance of bone grafting may provide a more significant advantage for patients with early implantation.

### Limitations of the study

For the reason that most antibodies are anti-rabbit, the present study lack immunofluorescence staining to directly determine the exact type of immune cells. Instead, it focuses on immune cells as one concurrent group. In addition, we did not strictly distinguish which inflammatory cells came from the PRF itself and which from the recruitment process. Future research should focus on discovering which types of immune cells are more strongly activated by PRF at early time points following sinus grafting precisely.

## Conclusion

The present study found that H-PRF bone blocks led to early infiltration of immune cells as early as 3 days post-surgery, leading towards an accelerated formation of new blood vessels and bone remodeling. Future research is needed to further investigate and optimize the protocols and ratios used of solid-PRF and liquid-PRF with various types of bone grafts used during sinus grafting procedures. Nevertheless, these early findings support recent clinical findings and trends for combining PRF into bone grafting procedures.

## Data Availability

The datasets used and analysed during the current study are available from the corresponding author on reasonable request.
